# A simulation based large bus side slip and rollover threshold study in slope-curve section under adverse weathers

**DOI:** 10.1371/journal.pone.0256354

**Published:** 2021-08-19

**Authors:** Lin Tian, Yanfei Li, Jueshuai Li, Wenzhen Lv

**Affiliations:** 1 School of Civil Engineering, Yantai University, Yantai, Shandong, China; 2 Chang’an University, Xi’an, China; Tongii University, CHINA

## Abstract

To study the side slip and rollover threshold of large bus in slope–curve section under adverse weather, factors that affect the safety of large buses that run in slope–curve section, such as rain, snow, cross-wind environmental factors, and road geometry, were analyzed to obtain the friction coefficient of the road surface under different rainfall and snowfall intensities through field measurements and to determine the six-component force coefficient of wind that acts on large buses through wind tunnel tests. The force analysis of large bus in slope-curve section was carried out, and the mechanical equations of large bus under the limit conditions of sideslip and rollover in slope-curve section were established. TruckSim simulation test platform was used to establish a three-dimensional road model and large bus mechanical model at a design speed of 100 km/h. Input parameters, such as cross-wind speed and road friction coefficient, simulate the impact of wind-rain/snow coupling. Under the combined action of wind-rain/snow, the operation test of large bus in slope-curve section was carried out, and the key parameters and indicators of the sideslip and rollover of large bus in slope-curve section were outputted and analyzed. The sudden change point of lateral acceleration is the judging condition for sideslip of large bus in slope-curve section under different road friction coefficient (0.2–0.7), changing from 0.15m/s^**2**^ and stabilizing to 0.52 m/s^**2**^, and a 0N vertical reaction force of the inner tire is the critical judging condition for rollover under road friction coefficient0.8, and the operating speed thresholds were proposed under different road friction coefficient. This study is expected to provide theoretical support for the speed limit of large bus in slope-curve section under adverse weather.

## 1. Introduction

In the statistics of road traffic accidents, the proportion of traffic accidents caused by large buses is increasing, and the fatalities and property losses caused by large buses are huge. Many large bus accidents are caused by sideslip and rollover in slope-curve section. The slope-curve section is a mixture of a small radius plane line and an uphill or downhill vertical section line, thereby causing the characteristic road section to become accident-prone section. In traffic accident statistics, the total number of deaths due to large bus accidents each year accounts for 65% of the total number of deaths from all vehicle-related accidents, and the proportion of fatalities caused by the sideslip and rollover of large passenger cars on curved and sloped road sections is 45% [1, 2]. These traffic accident cases show that large bus are prone to rollover traffic accidents in slope-curve section that cause heavy casualties and property losses. In recent years, the global climate environment has deteriorated because of the frequent occurrences of severe weather, such as wind, rain, and snow. Adverse weather has seriously affected road traffic safety. Under the influence of adverse weather, wind, rain, and snow, the risk of side slip and rollover accidents of large bus in slope-curve section has increased. When large buses run at higher speeds in slope-curve section under the action of strong cross wind, the driving stability and handling and car characteristics will be reduced significantly because of their large body volume, large side windward area, and high center of mass. Ride comfort, and even side-slip or rollover accidents, seriously affect the operation safety of large buses. Rain or snowfall reduces the friction coefficient of the road surface significantly and increases the risk of side slips and rollovers of large bus. Thus, the operation safety of large bus in slope-curve section under adverse weather needs to be solved urgently.

In terms of large bus running stability, the composite material was applied to the rigidity and strength requirements of bus roll bar to improve the stability of rollover [3]. The severity of bus accidents was evaluated by fitting a logit mode, and the rollover and slippage of bus accidents are related to factors, such as vehicle size, running speed, road alignment, weather conditions, and driving time [4–7]. Establishing a vehicle system control model based on typical four-wheel steering and direct yaw moment control methods was proposed to improve bus rollover stability [8–11]. In terms of road geometry safety, some scholars analyzed the force characteristics of cars under different road geometry conditions and established the mathematical models of car sideslip and rollover. Some scholars analyzed the force characteristics of cars under different road geometry conditions, established the mathematical models of car sideslip and rollover, and analyzed the impact of alignment on traffic safety [12–17]. Scholars have summarized the history of curve linear design in the United States from the two aspects (e.g., flat and vertical curves), especially the setting of easement curves [18, 19]. In terms of traffic safety in adverse weather, scholars obtained basic data on the operation of high-side bus under crosswinds through wind tunnel tests of 1:50 car models and studied the aerodynamic response of crosswinds to trucks [20–22]. Scholars also analyzed the relationship between the rain accident rate and the lock brake coefficient. The conclusion showed the nonlinear relationship between the accident rate on wet roads and anti-skid performance [23]. Statistically, scholars analyzed the accident data of different types of roads, and the results showed that the accident rate increased significantly with the decrease in road adhesion, and the accident rate had a linear function relationship with the anti-skid performance of the road surface [24–26]. Studies have shown that wet and slippery roads on rainy days increase the incidence of accidents, and the occurrence of collision accidents is mostly due to the driver’s own reasons, such as inattention, and less due to the geometric characteristics of the road [27, 28]. Domestic and foreign scholars have carried out a large number of independent studies on the stability of vehicle side slip and rollover, road alignment, road traffic safety under adverse weather, and vehicle aerodynamics. Studies on the safety of automobile operation under the combination of adverse weather and slope–curve are limited.

The mechanical model of the large bus in slope-curve section was established, and the speed model for the safe operation of the large bus under extreme conditions was derived through the force analysis of the large bus in slope-curve section. The environmental risk factors of rain, snow, and crosswinds are tested to obtain the friction coefficient of the road surface under adverse weather and determine the six-component force coefficient of wind that acts on large buses through wind tunnel tests. The TruckSim simulation platform was used to establish a three-dimensional (3D) road model with a design speed of 100 km/h and a 3D mechanical model of a large bus. The friction coefficient of the road surface and the wind speed of cross wind in adverse weather are inputted, and simulation tests are conducted under different combinations of working conditions to identify the conditions for side slip and rollover of a large bus and to determine the speed threshold for the large bus to cause sideslip and rollover.

## 2. Mechanical analysis of the operation of large bus in slope-curve section

### 2.1 The rollover mechanics model of large passenger bus in slope-curve section

**Fig 1:**
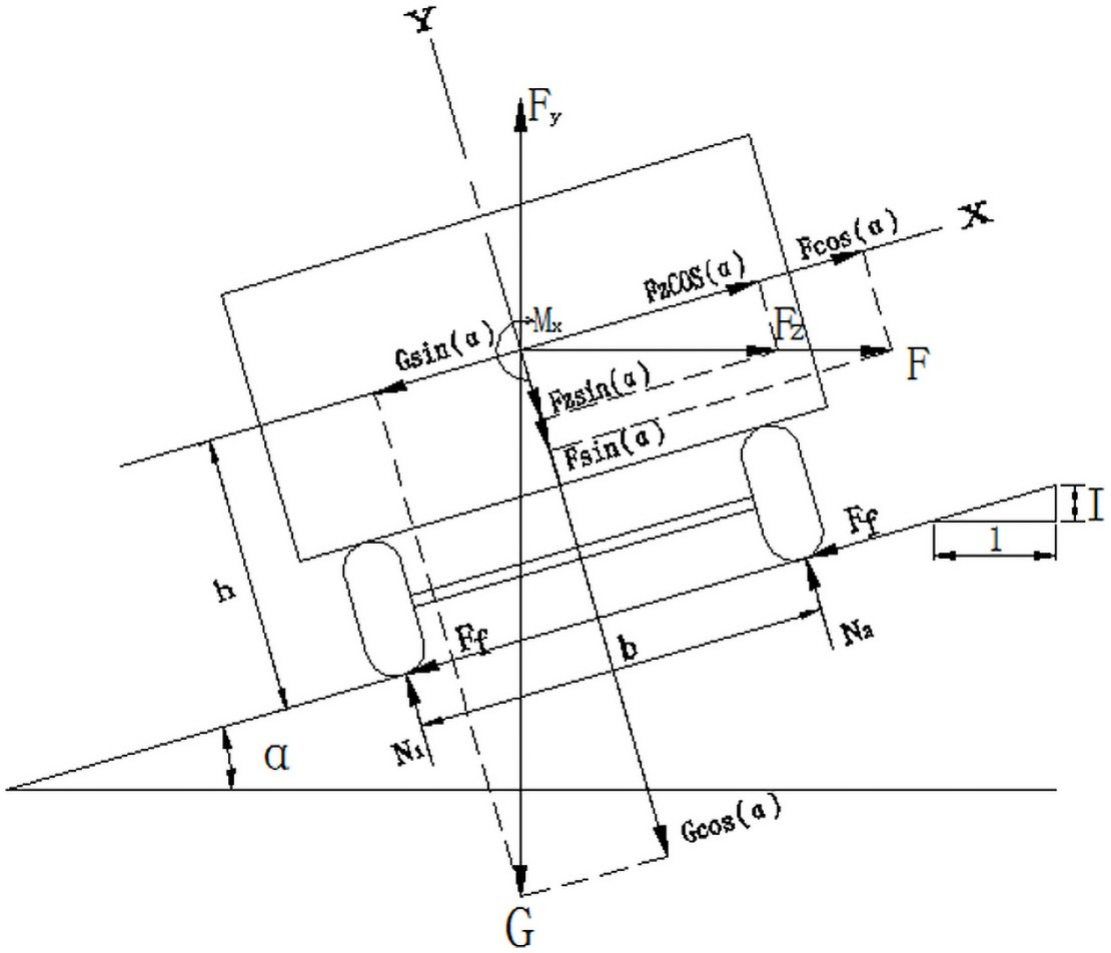
Stress analysis of bus in curve sections. *F*_f_—Road friction (KN); *N*_1_, *N*_2_—Road support force (KN).

In the force analysis of large buses, the elastic deformation of the suspension and tires of a car is ignored, and the car is assumed to be a rigid body [29]. The force analysis of the large bus in slope–curve section is carried out (Fig 1).

The lateral force *G*, aerodynamic lift *F*_*y*_, pneumatic lift *F*_*z*_, and centrifugal force *F* generated by the gravity and cross wind of the large bus are decomposed into the lateral force *X* parallel to the road surface and the vertical force *Y* perpendicular to the road surface, namely:

$$X=F\text{cos}\alpha +F_{y}\text{cos}\alpha -G\text{sin}\alpha$$ (1)

$$Y=F\text{sin}\alpha +F_{y}\text{sin}\alpha +G\text{cos}\alpha -F_{z}$$ (2)

The cross slope angle of the road surface is generally small. Thus, sin *α* = tan *α* = *I*, and cos *α* ≈ 1, among them, the composite slope of the transverse superelevation value *i*_h_ and the longitudinal slope of the curved slope *i* combination section $I=\sqrt{i^{2}-i_{h}^{2}}$, and so:

$$X=F+F_{\text{y}}-GI$$ (3)

$$Y=FI+F_{\text{y}}I+G-F_{\text{z}}$$ (4)

Lateral force is an unstable factor for large bus, and vertical force is a stable factor. During the operation of the car under the action of cross wind, the car runs stably when the stable torque is greater than the rollover torque. FI and FyI relative to the large bus’s gravity G are very small and can be ignored. Then, the rollover mechanics model can be simplified as:

$$\left(mg-F_{z}\right)\frac{b}{2}\geq \left(\frac{mv^{2}}{R}+F_{y}-mgI\right)h$$ (5)

The calculation model of the running speed of a large bus traveling in slope–curve section without rollover is:

$$v\leq \sqrt{127R\left(\frac{b}{2h}+I-\frac{F_{y}}{G}-\frac{F_{z}}{G}\cdot \frac{b}{2h}\right)}$$ (6)

where R is the radius of the circular curve (m), b is the wheelbase of the car (m), h is the height of the car’s center of mass (m), v is the driving speed of the car (km•h − 1), and others are the same as above.

### 2.2 Mechanical model of side slip of large bus in slope-curve section

When a car is driving in slope–curve section, the car may produce lateral slip in the direction of the lateral force because of the presence of lateral force. The lateral force is less than or equal to the lateral frictional resistance between the tire and the road surface, namely:

$$X\leq Y\varphi_{h}\approx \left(G-F_{z}\right)\varphi_{h}$$ (7)

$$\frac{Gv^{2}}{gR}+F_{y}-GI\leq \left(G-F_{z}\right)\varphi_{\text{h}}$$ (8)

where *φ*_*h*_ is the friction coefficient of the road surface.

The calculation model for the running speed of a bus driving in slope–curve section without sideslip is:

$$v\leq \sqrt{127R\left(\varphi_{h}+I-\frac{F_{y}}{G}-\frac{F_{z}}{G}\cdot \varphi_{h}\right)}$$ (9)

Formulas (6) and (9) are the mechanical models for the large bus to avoid sideslip and rollover in slope-curve section, respectively. The large bus satisfies both formulas during the operation in slope–curve section to ensure that no side slip and rollover accidents occur.

## 3. Simulation test of large bus in slope-curve section

### 3.1. Test and quantification of adverse weather influence factors

**Fig 2:**
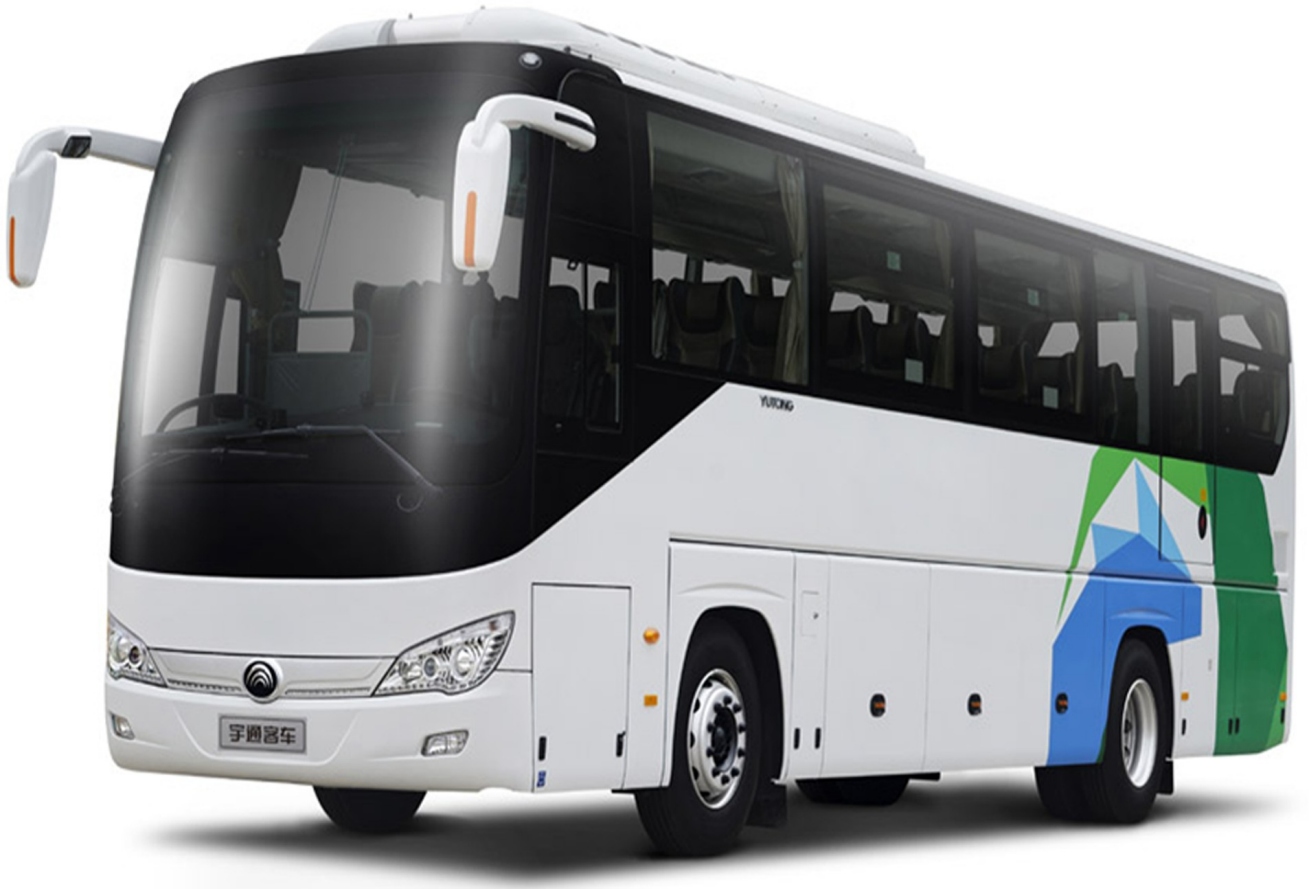
Test leading model.

The adverse weather effects that affect large bus in slope-curve section were quantified, to obtain the aerodynamic coefficient of large bus through wind tunnel test, and to obtain the road friction coefficient through field test in rain and snow. A large bus of a certain model (YUTONG-ZK6117BEV) was selected as the test model (Fig 2), and its detailed parameters are shown in Table 1. This model was selected as the leading model for the test mainly because of its large operating ratio in large bus, large side windward area, high center of mass, high inertia during operation, and high traffic accident severity.

**Table 1:** Performance parameters of leading model.

bus body height (mm)	3450
bus body width (mm)	2550
Bus body length (mm)	10990
distance between bus body center of mass and front axle (mm)	3704
distance between car body center of mass and rear axle (mm)	2945
sprung mass (kg)	12250
rolling inertia of car body (kg·m^2^)	14823
ritching inertia of car body (kg·m^2^)	59290

#### (1) Six-component coefficient of large bus

The determination of the aerodynamic six-component force of the test model in the wind tunnel test was achieved by simulating the ambient wind with the aid of the yaw angle of the test model relative to the airflow direction. The wind tunnel test conditions were detailed as follows: the test angle of the attack is 0° and 90°, and wind tunnel tests were carried out under two conditions. To eliminate the interference of the stent to the aerodynamic characteristics of the test model, the test must be repeated to obtain and deduct the individual aerodynamic characteristic data of the stent.

In the horizontal uniform wind field, the test angle of the attack was changed, and the test wind speed is 13 m/s (sixth wind speed). The measurement results of the aerodynamic force coefficient of the vehicle according to the six and aerodynamic force coefficients are shown in Table 2. The formula can be used to calculate the aerodynamic force and moment of a large bus under the action of cross winds with different wind speeds.

**Table 2:** Six-component coefficient of large bus.

wind speed/m·s^-1^	angle of attack/°	*C*_*D*_	*C*_*L*_	*C*_*S*_	*C*_*Mx*_	*C*_*My*_	*C*_*Mz*_
13	0	0.046283	0.026795	0.784652	0.062253	-0.005087	0.026049
	90	0.043772	0.025044	0.766509	0.062253	-0.003213	0.012438

#### (2) Road friction coefficient under rain and snow conditions

**Fig 3:**
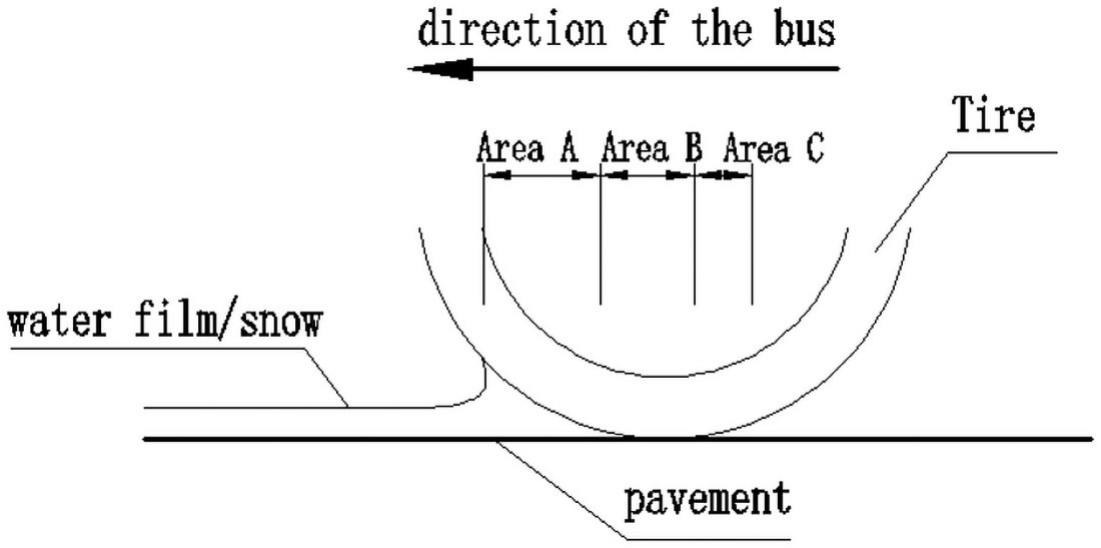
Schematic diagram of tire contact with road surface.

Under rain condition, rainwater forms a water film on the road surface. When a car is driving on a road with water, the water will reduce the contact area between the tire and the road. The reduced contact area is the basic area of the tire and the water film. The contact surface between the roads can be divided into three areas, as shown in Fig 3.

Area A was a completely floating water area, where the water pressure was sufficient to lift the thread and make it completely separated from the road surface. A large amount of water disperses on the road surface in Zone B, and water was found in the concave part of the road surface, and the convex part has formed a dry area, which is called an incomplete contact area. Almost all of the water in zone C is squeezed out, which is close to a dry state, and is the area where the tread is completely in contact with the road surface. Adhesion is mainly produced in zone C, followed by zone B. Zone A does not only not produce adhesion but also lifting force on the wheels to make the wheels float.

Through the test of the friction coefficient of the road pendulum instrument under the conditions of rain and snowfall, the friction coefficient of the road surface is 1/2 of that of the normal dry road surface, which is reduced to approximately 0.5. Under snow conditions, the friction coefficient of snow or icy roads is only 1/8 to 1/4 of that of normal dry roads, 0.1 for icy roads, and 0.2 for snowy roads.

### 3.2. Establishment of a three-dimensional road model of slope-curve section

**Fig 4:**
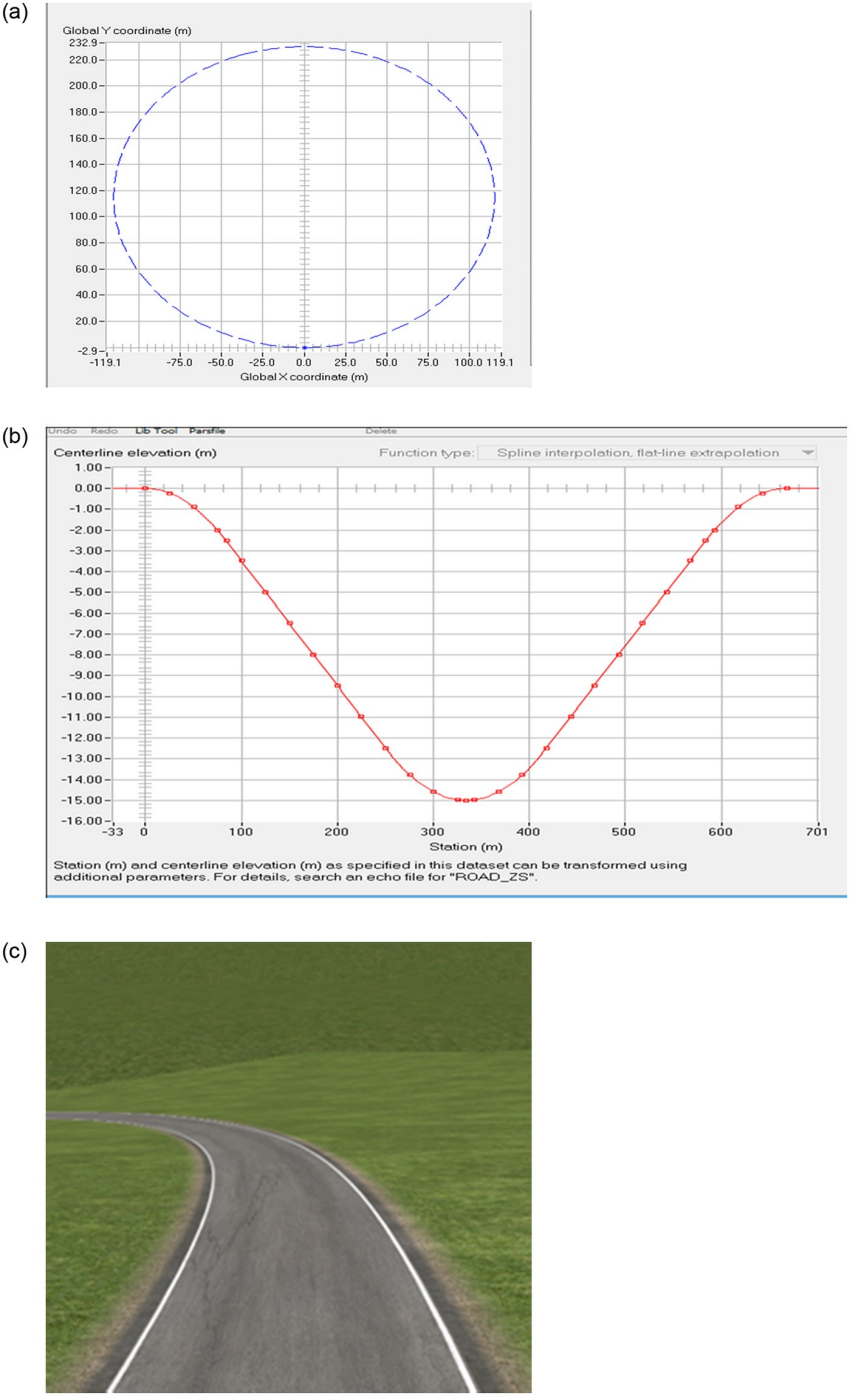
Three-dimensional road model of slope-curve section of TruckSim.

In this paper, carrying out the actual vehicle test of the side slip and rollover limit condition in slope-curve section is impossible. The TruckSim simulation test is used to simulate the wind-rain/snow coupling effect to download the side slip and rollover accident form of the heavy vehicle in slope-curve section. In the TruckSim simulation platform, 3D splines are used to establish curved and slope composite road sections. By defining the (X, Y, Z) 3D coordinates of the road surface center point, the radius of the road curve, as shown in Fig 4(a),the road longitudinal slope, as shown in Fig 4(b), and the super high cross slope, and the road surface friction coefficient are inputted. A 3D spline road model is established, and the currently established road model of in slope-curve section, as shown in Fig 4(c). The technical indicators of the test section are based on the "Design Specification for Highway Alignment" (JTG D20-2017) [30]. The minimum limit value of the circular curve with a design speed of 100 km/h is selected as 400 m, the superelevation value of the cross section is 8%, and the slope of the vertical section is 5%. The calculated slope is 9.43%.

### 3.3. Establishment of three-dimensional mechanical model of large bus

**Fig 5:**
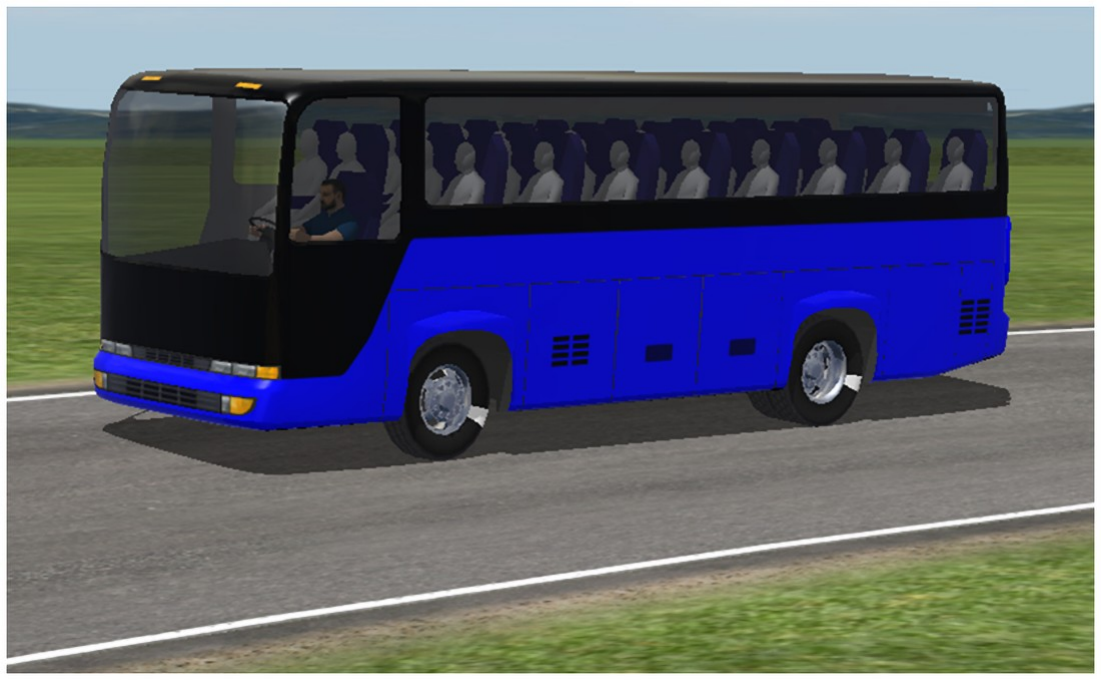
Three-dimensional mechanical model of large bus of TruckSim.

TruckSim simulation software is used to model the front suspension, rear suspension, steering system, power system, tires and body of the car. The communicator is utilized to establish the mutual transfer of data between the subsystems, templates, and test benches, transfer each component, and assemble and build a complete vehicle model (Fig 5). A simulation model is established to simplify the automobile system to a certain extent and present in the form of a mathematical model.

### 3.4. Combined simulation input of adverse weather effects

The wind level that acts on the large bus is the sixth wind, the wind speed is 13.8 m/s, and the wind direction is perpendicular to the side of the large bus body. The friction coefficient of the road surface under rainfall conditions is 0.4 and 0.5, and the friction coefficient of the road surface under snow conditions is 0.3 and 0.2. In good weather conditions, the pavement resistance coefficient is taken as 0.8. The combination of adverse weather, wind and rain, wind and snow, and wind alone are imported into the TruckSim simulation platform to simulate the impact of adverse weather combinations on large bus.

## 4. Results

### 4.1. Simulation of side-slip conditions of large bus in slope-curve section

**Fig 6:**
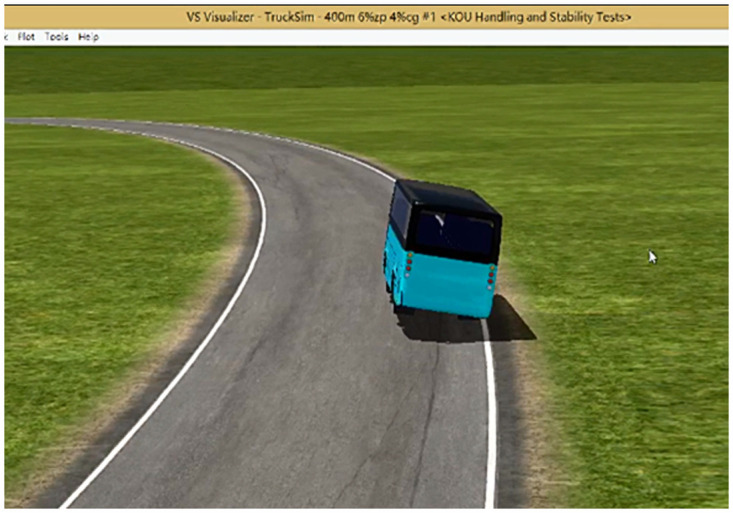
Critical state of large bus sideslip.

The six-level wind speed is 13.8 m/s, and the road surface friction coefficient is 0.2, 0.3, 0.4, 0.5, 0.6, and 0.7. In the simulation test of a large bus under the combination of working conditions, the critical state of side slip is shown in Fig 6, and the large bus has lateral slip.

### 4.2. Simulation of rollover conditions of large bus in slope-curve section

In the simulation test of a large bus under the combination of a six-level wind speed of 13.8 m/s and a road friction coefficient of 0.8, the critical speed for a large bus to rollover is 152 km/h.

## 5. Discussion

The range of road friction coefficient is 0.2–0.4, which simulated the road conditions in poor snow weather. The range of road friction coefficient is 0.4–0.6, which simulated the road conditions under rain conditions. The range of road friction coefficient is 0.6–0.8, which simulated the weather. The road condition is good.

**Fig 7:**
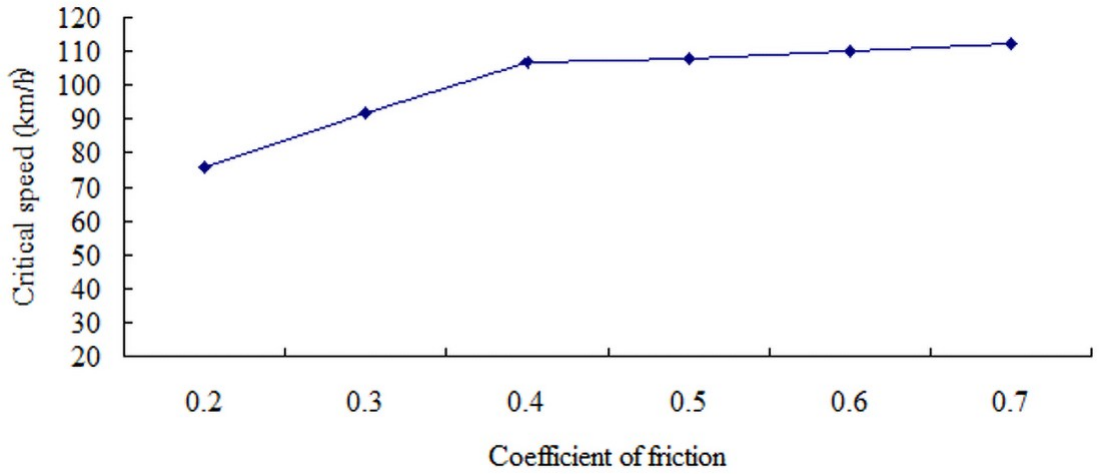
Graph between critical speed and coefficient of friction of sideslip.

The sudden changes in lateral acceleration that correspond to the road friction coefficients of 0.2, 0.3, 0.4, 0.5, 0.6, and 0.7 are shown in Table 3. The critical speed for the side slip of a large bus increases with the friction coefficient, as shown in Fig 7. Under rain or snow conditions, the speed at which the large bus does not sideslip in the test slope–curve section should be lower than the speed threshold listed in Table 3.

**Table 3:** Critical speed type with sideslip.

Coefficient of friction	0.2	0.3	0.4	0.5	0.6	0.7
Critical speed (km/h)	76	92	107	108	110	112
Lateral acceleration (m/s^2^)	0.15	0.17	0.31	0.32	0.45	0.52

**Fig 8:**
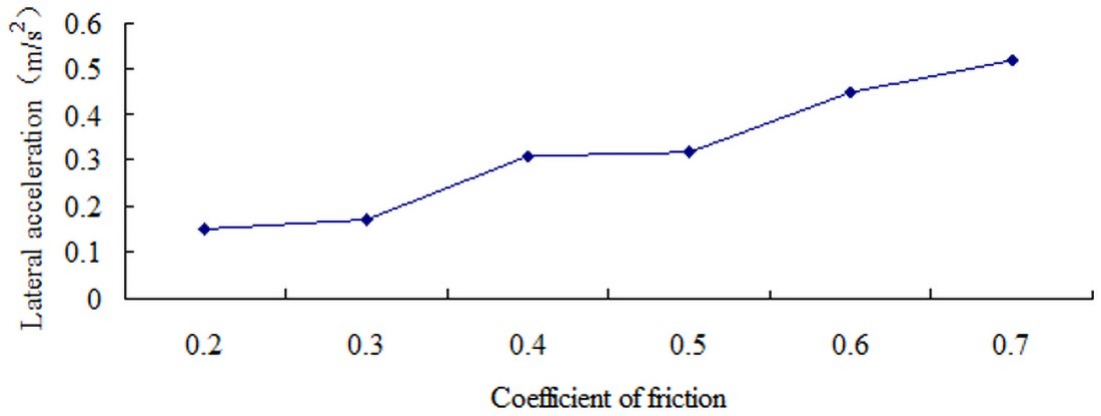
Graph between lateral acceleration and coefficient of friction of sideslip.

**Fig 9:**
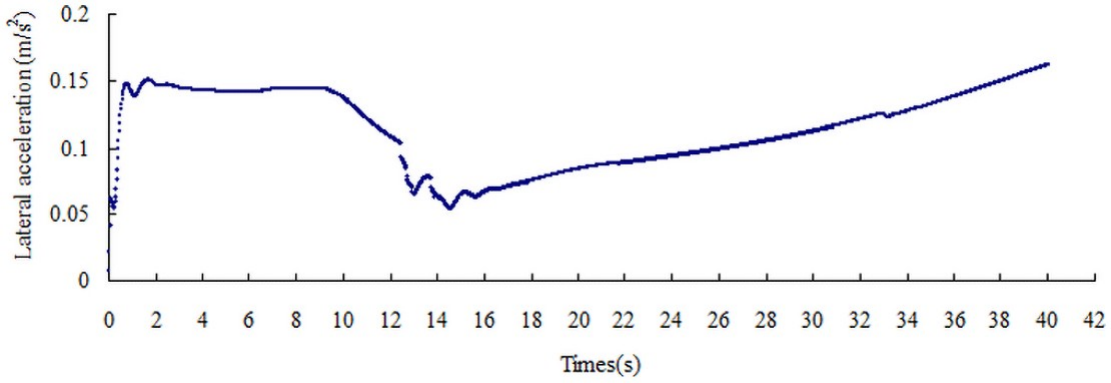
Lateral acceleration change graph of friction coefficient 0.2.

**Fig 10:**
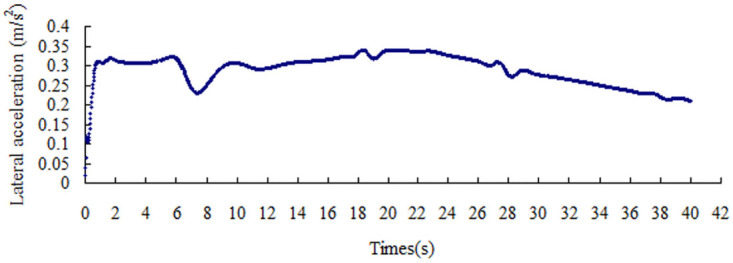
Lateral acceleration change graph of friction coefficient 0.3.

**Fig 11:**
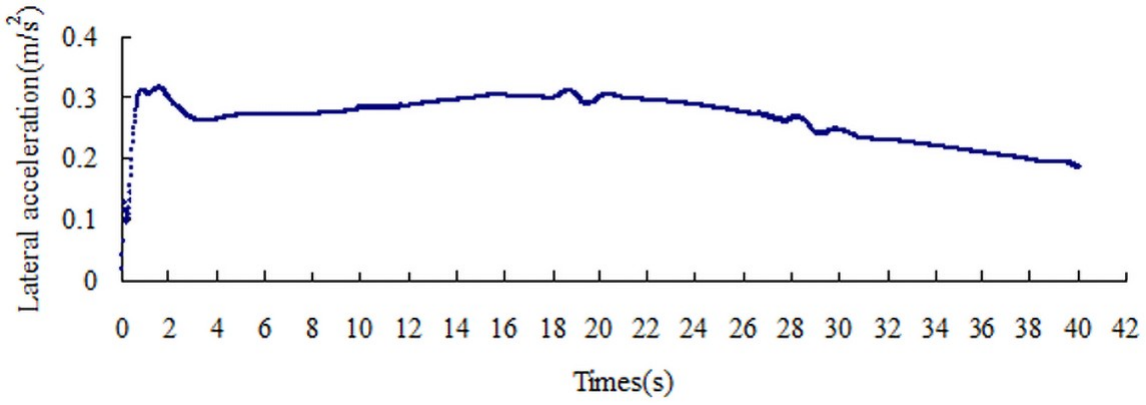
Lateral acceleration change graph of friction coefficient 0.4.

**Fig 12:**
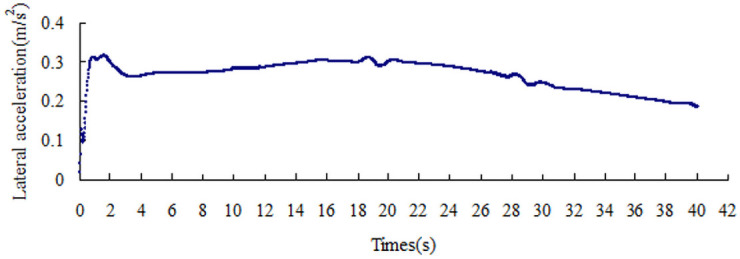
Lateral acceleration change graph of friction coefficient 0.5.

**Fig 13:**
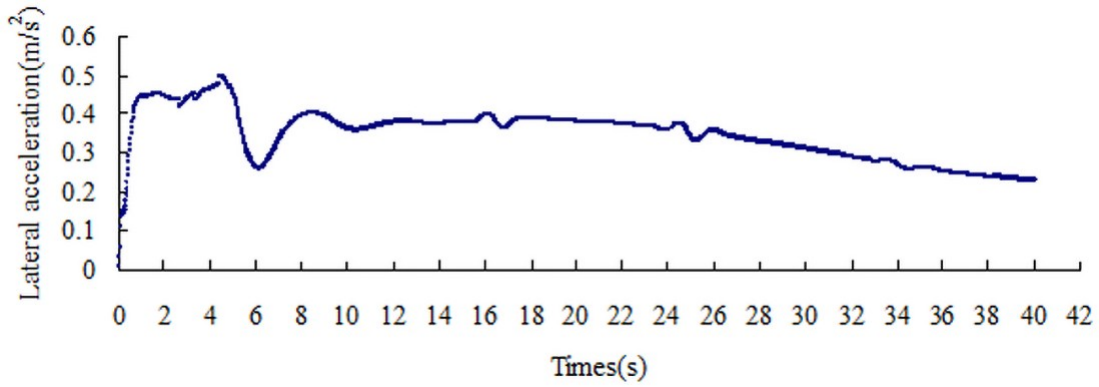
Lateral acceleration change graph of friction coefficient 0.6.

**Fig 14:**
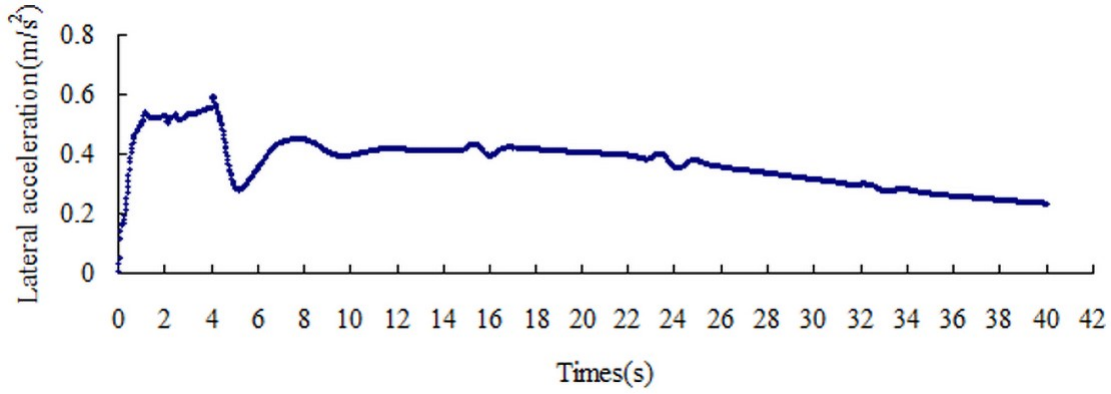
Lateral acceleration change graph of friction coefficient 0.7.

The lateral acceleration gradually increases with the increase in the friction coefficient, that is from 0.15 m/s^2^ to 0.51 m/s^2^, as shown in Figs 8–14. The identification index for the side slip of a large bus is the sudden change in lateral acceleration, as shown in Figs 9–14. In adverse weather rain and snow, the friction coefficient of the road surface decreases, thereby making the large bus prone to side-slip accidents. The lower the road friction coefficient is, the greater the risk of side-slip will be.

**Fig 15:**
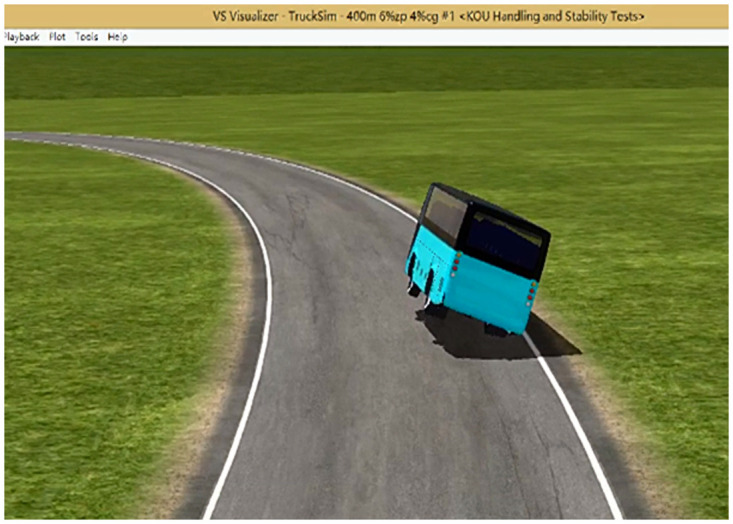
Critical state of large bus rollover.

**Fig 16:**
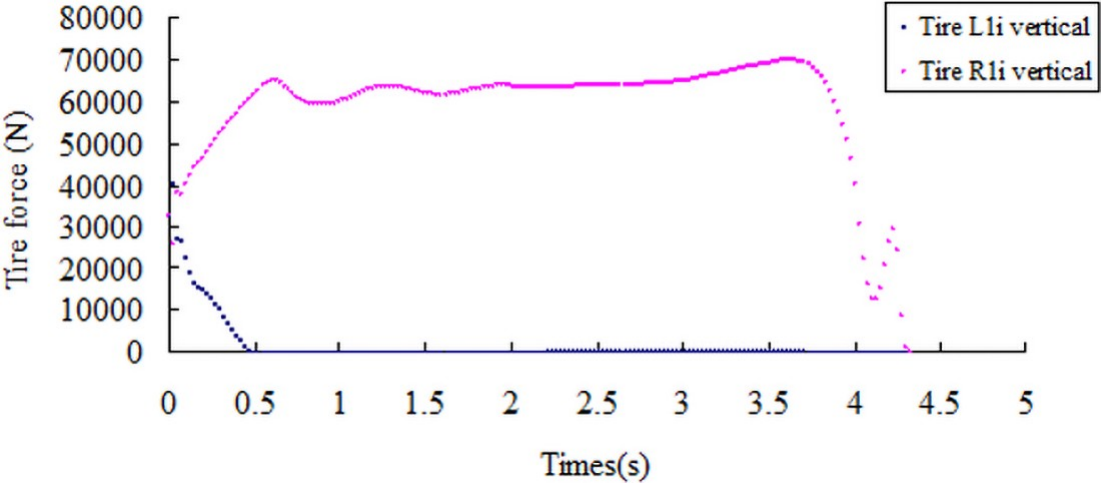
Vertical reaction curve of the front axle tire with a friction coefficient of 0.8.

**Fig 17:**
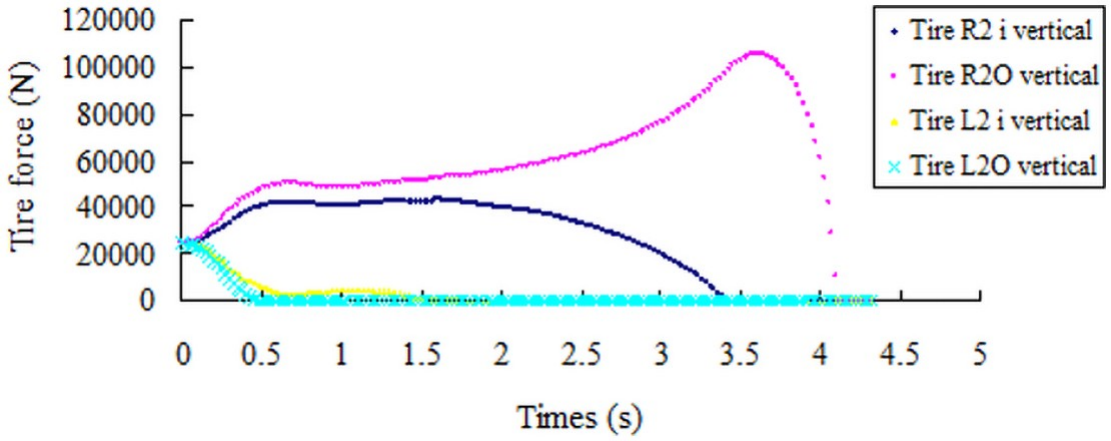
Vertical reaction curve of the rear axle tire with a friction coefficient of 0.8.

The critical state of the rollover is shown in Fig 15. In the screening index, the vertical reaction force diagram of the left tire of the front and rear axle becomes 0 N, the vertical reaction force of the right tire increases abruptly, and the change curve of the vertical reaction force of the left and right tires is shown in Figs 16 and 17. Through simulation tests, the road friction coefficient is in the range of 0.2–0.7, large bus only experience sideslip accidents, and the road friction coefficient is greater than 0.7, and only rollover accidents occur.

## 6. Conclusion

The mechanical analysis of the critical conditions of rollover and sideslip of large bus slope–curve section was carried out, and the effects of poor weather, wind, rain, and snow were introduced. A speed model for the limit conditions of rollover and side slip of large bus in the slope–curve section was established, as shown in calculation models (6) and (9). Through the TruckSim simulation test, the critical condition for judging the sideslip of the large bus in the slope–curve section is determined to be the lateral acceleration sudden change point, and the critical condition for the rollover is that the vertical reaction force of the inner wheel is reduced to 0 N. The TruckSim simulation test obtained that the speed threshold of the large bus without sideslip under the combination of 6-level wind speed of 13.8 m/s and different friction coefficients (0.2, 0.3, 0.4, 0.5, 0.6, and 0.7) is 76 km/h, 92 km/h, 107 km/h, 108 km/h, 110, and 112 km/h, respectively. The sixth wind speed is 13.8 m/s, the road friction coefficient is 0.8, and the speed threshold for the rollover is 152 km/h. Theoretical support for the safety speed limit standard of large passenger vehicles in the slope–curve section under adverse weather is provided, and the safety level of large passenger vehicles in the slope–curve section under adverse weather are improved.
